# Spreading dynamic of acute and carrier hepatitis B with nonlinear incidence

**DOI:** 10.1371/journal.pone.0191914

**Published:** 2018-04-05

**Authors:** Tahir Khan, Gul Zaman, Ali Saleh Alshomrani

**Affiliations:** 1 Department of Mathematics, University of Malakand, Chakdara Dir (Lower), Khyber Pakhtunkhawa, Pakistan; 2 Department of Mathematics, Faculty of Science, King Abdul Aziz University, Jeddah, Saudi Arabia; Kliniken der Stadt Köln gGmbH, GERMANY

## Abstract

Hepatitis B infection caused by the hepatitis B virus. It is one of the serious viral infection and a global health problem. In the transmission of hepatitis B infection different phases, i.e., acute and chronic carrier stages play an important role. The chronic carries individuals do not exhibit any symptoms and are able to transmit the infection. Here we assessed the transmissibility associated with different infection stages of hepatitis B and generated an epidemic model with nonlinear incidence rate. In order to do this, first we formulate the model by splitting the infectious class into two subclasses, namely acutely infected and chronic carries with both horizontal and vertical transmission. The basic properties of the proposed model are presented. The basic reproductive number is obtained by using the next generation matrix approach. Biological sense of the threshold condition is investigated and discussed in detail. We also find the conditions to investigate all possible equilibria of the model in terms of the basic reproduction number. Finally, we perform numerical simulations to support our analytical work.

## Introduction

Hepatitis B has different phases, such as acute and chronic carrier hepatitis. Acute hepatitis B refers to the first six months after exposed some one to hepatitis B virus. In this stage the immune system is usually able to clear the virus from the body and some one of the individual may be recovered within few months complectly. However, the remaining infection grows and leads to more serious stage called chronic stage or life long illness. Chronic carrier hepatitis B virus stays for long time inside the body and develop serious health problem. Individuals with carrier hepatitis often have no history of acute illness, but it can cause liver scarring which becomes the cause of liver failure [1].

Hepatitis B is a globally health problem and a leading cause of death in the world. Million of people are infected with hepatitis B virus infection among them about 2 millions individuals live chronically. So from these infected individuals including both chronic or acute every year approximately 780, 000 people dies [2]. Medical researchers also investigated that hepatitis B virus infection is responsible for about 80 percent of the primary liver cancer.

The virus can be transmitted in the community from one individual to another in different ways including sharing syringes, blood transfusion and unprotected sexual contacts etc. It is also transmitted to a new born baby from her infected mother at the time of birth known is vertical transmission. Most infected adults carry the capacity to overcome hepatitis B virus, however some adults and children are extremely vulnerable to this virus and develop acute infections. Although there is a vaccine and new techniques available in the market to prevent the transmission of hepatitis B virus, but recently still new cases are reported.

Several biologists and mathematicians have been developed different epidemic models for the transmission dynamic of infectious diseases in the population [3–15]. Currently different mathematical models have been developed to understand the transmission dynamics of hepatitis B in the community [1, 16, 17]. The incidence rate is one of the key concept and plays an important role in the study of mathematical modeling. The nonlinear incidence rate is more reasonable then bilinear incidence rate, especially in case of sexually transmittible diseases like hepatitis B and HIV etc. Bilinear incidence rate *βSI* frequently used in many epidemic models [18, 19], where *β* represents the contact rate, *S* represents susceptible individuals and *I* represents the infectious individuals. Nonlinear incidence rate $\frac{\beta SI}{N}$ used in [20], where *β* is the contact rate, *S*, *I* are susceptible, infected respectively and *N* represents the total population.

In this article, we develop a hepatitis B virus transmission model by incorporating the acute and chronic carrier infected subclasses with nonlinear incidence rate in the host population. In order to consider the acute infected and chronic carrier individuals with nonlinear incidence rate in the host population, the total population is divided into six epidemiological classes and retrieve a new mathematical model. First, we show the basic properties like positivity, boundedness and biological feasibility. Then, the reproduction number is investigated and discuss its sensitivity analysis. We also discuss the stability analysis at both disease free and endemic equilibrium by using linearzation, Lyapunov function theory and geometrical approach. Finally, results of numerical simulations are presented.

The paper is organized as follows: First section is devoted to introduction. In the second section, we formulated the proposed hepatitis B model. In the third section, we discussed the existence of positive solution and biologically feasibility of the proposed model. In fourth section, we obtained the basic reproductive number and discussed its sensitivity analysis. The fifth section is concerned to the stability analysis of the model. Finally in the sixth and seventh sections, numerical simulation and conclusion are presented, respectively.

## Formulation of the hepatitis B model

In this section, we develop a hepatitis B virus transmission model with nonlinear incidence rate. According to the biological characteristic of hepatitis B virus, the total population *N*(*t*), is divided into six epidemiological subclasses, namely susceptible *S*(*t*), latent *L*(*t*), acutely infected *A*(*t*), chronic carrier infectious *C*(*t*), recovered with permanent immunity *R*(*t*) and vaccinated *V*(*t*). Keeping the characteristic of hepatitis B, we place the following assumptions on the model:

*Y*_1_. The initial populations *S*(0), *L*(0), *A*(0), *C*(0), *R*(0) and *V*(0) are all known and non-negative.
*Y*_2_. The inflow of new born with parentally infection goes to carrier compartment.
*Y*_3_. The inflow of new born without parentally infection goes to susceptible compartment.
*Y*_4_. The population with successful vaccination goes to the vaccinated compartment.
*Y*_5_. Recovered individuals has a permanent immunity.
*Y*_6_. The inflow of new born with successful vaccination goes to vaccination compartment.

The assumptions *Y*_1_−*Y*_6_ lead to a mathematical model represented by the following system of seven differential equations:

$$\begin{array}{c} \begin{array}{cc} \frac{dS(t)}{dt} & =b\xi N\left(1-\eta C\left(t\right)\right)+\phi V\left(t\right)-\frac{\beta S(t)A(t)}{N}-\frac{\gamma \beta S(t)C(t)}{N}-\left(d_{0}+\gamma_{3}\right)S\left(t\right),\\ \frac{dL(t)}{dt} & =\frac{\beta S(t)A(t)}{N}+\frac{\gamma \beta S(t)C(t)}{N}-\left(d_{0}+\sigma \right)L\left(t\right),\\ \frac{dA(t)}{dt} & =\sigma L\left(t\right)-\left(d_{0}+\gamma_{1}\right)A\left(t\right),\\ \frac{dC(t)}{dt} & =b\xi \eta NC\left(t\right)+p\gamma_{1}A\left(t\right)-\left(d_{0}+d_{1}+\gamma_{2}\right)C\left(t\right),\\ \frac{dR(t)}{dt} & =\gamma_{2}C\left(t\right)+\left(1-p\right)\gamma_{1}A\left(t\right)-d_{0}R\left(t\right),\\ \frac{dV(t)}{dt} & =bN\left(1-\xi \right)+\gamma_{3}S\left(t\right)-\left(d_{0}+\phi \right)V\left(t\right), \end{array} \end{array}$$ (1)

with initial conditions

$$\begin{array}{c} S(0)>0,L(0)\geq 0,A(0)\geq 0,C(0)\geq 0,R(0)\geq 0,V(0)>0. \end{array}$$ (2)

In the model (1), *b* represents the birth rate, *ξ* represents the birth rate without successful vaccination, *η* represents the proportion of prenatally infected individuals, *ϕ* shows the rate of waning vaccine induced immunity, *β* shows the transmission rate from susceptible to infected, *γ* represents the reduced transmission rate. *d*_0_ represents the death rate, which occur naturally. *γ*_3_ represents the vaccination rate. *σ* represents the moving rate from latent class to acute class. *γ*_1_ represents the moving rate from acute to chronic carrier. *γ*_2_ represents the moving rate of chronic carrier to immune. *d*_1_ represents the death rate, which occur from the hepatitis B and *p* represents the average probability of those individuals, who fail to recover in acute stage and goes to chronic carrier.

## Basic mathematical properties

**Proposition 1**. *For all t* > 1 *and the initial data S*(0) > 0, *L*(0) ≥ 0, *A*(0) ≥ 0, *C*(0) ≥ 0, *R*(0) ≥ 0 *and V*(0) > 0, *the solution* (*S*, *L*, *A*, *C*, *R*, *V*) *of model* (1) *are positive, whenever they exist*.

**Proof**: Let *φ*_1_ = 1 − *ηC*(*t*), $\varphi_{2}=\frac{\beta A(t)}{N}+\frac{\beta \zeta C(t)}{N}$ and *I* ⊂ [0, + ∞), then the first equation of system (1) can be written as

$$\begin{array}{c} \frac{dS(t)}{dt}=b\xi N\varphi_{1}+\phi V\left(t\right)-\left(d_{0}+\gamma_{3}+\varphi_{2}\right)S\left(t\right). \end{array}$$ (3)

The assumption that the solution of the system (1) exists in the interval *I*, for all *t* ∈ *I*, the solution of Eq (3) look likes

$$\begin{array}{c} \begin{array}{cc} S(t) & =S\left(0\right)\text{exp}\{-(\left(d_{0}+\gamma_{3}\right)t+\int_{0}^{t}\varphi_{2}\left(x\right)dx)\}\\ & +\text{exp}\{-(\left(d_{0}+\gamma_{3}\right)t+\int_{0}^{t}\varphi_{2}\left(x\right)dx)\}\\ & \times \int_{0}^{t}(b\xi N\varphi_{1}+\phi V\left(t\right))\text{exp}\{\left(d_{0}+\gamma_{3}\right)y+\int_{0}^{\ell }\varphi_{2}\left(u\right)du\}dy>0. \end{array} \end{array}$$ (4)

Obviously the right hand side of Eq (4) is positive. Consequently *S*(*t*) > 0 for all *t* ∈ *I*.

The solution of the second equation of model (1) yields

$$\begin{array}{c} L\left(t\right)=L\left(0\right)\text{exp}(-(\sigma +d_{0})t)+\text{exp}(-(\sigma +d_{0})t)\times \int_{0}^{t}\varphi_{2}\left(y\right)\text{exp}\left(\sigma +d_{0}\right)ydy\geq 0, \end{array}$$ (5)

which showing that *L*(*t*) ≥ 0. In a similar fashion, it can be shown that *A*(*t*) ≥ 0, *C*(*t*) ≥ 0, *R*(*t*) ≥ 0 and *V*(*t*) > 0. Hence the solution (*S*, *L*, *A*, *C*, *R*, *V*) of model (1) are positive for all *t* > 0, *t* ∈ *I*.

**Proposition 2**. *The system* (1) *is a dynamical system in the biological feasible region given by*

$$\begin{array}{c} \Omega =\{\left(S,L,A,C,R,V\right)\in R_{+}^{6}:N\left(t\right)\leq \frac{bN}{d_{0}}\}. \end{array}$$ (6)

**Proof**: The differentiability of the right hand side of the system (1) implies that the existence of the unique maximal solution for any associated cauchy problem. Thus the initial value problem (1) is well posed and biologically feasible, because all the state variables are non-negative. So for the required result, it is sufficient to study the dynamics of the flow generated by the system (1). Furthermore, since the solutions of the system (1) are positive and bounded, it remains to show that the vector field defined by this system is transversal to the boundary of *Ω* on all its faces. The face corresponding to $S=\frac{bN}{d_{0}+\gamma_{3}}$ has direction (1, 0, 0, 0, 0, 0) and the inner product with the vector field is *bξφ*_1_ + *ϕV*(*t*) − *βφ*_2_*S*(*t*) − (*d*_0_ + *γ*_3_)*S*(*t*) ≤ *bξφ*_1_ − (*d*_0_ + *γ*_3_)*S*(*t*). Similarly, we can check for the faces *L*, *A*, *C*, *R*, *V*. At last, the face corresponding to $N\left(t\right)=\frac{bN}{d_{0}}$ has a direction (1, 1, 1, 1, 1, 1) and the inner product with the vector field is *bN* − *d*_0_*N*(*t*) − *d*_1_*C*(*t*) ≤ *bN* − *dN*(*t*). Thus the vector field on these faces point toward the region Ω.

## Basic reproduction number analysis

### Computation of *R*_0_

In epidemiological models the role of basic reproduction number is a key concept and play a very important role. It represents the expected average number of new infections produced directly and indirectly by a single infective, when introduced into a completely susceptible population. To find the basic reproductive number for our proposed model (1), we follow Driessche and Watmough [21, 22]. Let us assume that, *χ* = (*x*_*i*_/*i* = 1…6)^*t*^, where *x*_1_ = *L*, *x*_2_ = *A*, *x*_3_ = *C*, *x*_4_ = *S*, *x*_5_ = *R*, *x*_6_ = *V* with each *x*_*i*_ ≥ 0. We also define *χ*_0_ to be the set of all disease free states, such that *X*_0_ = {*χ* ≥ 0/*x*_*i*_ = 0, *i*…*m*}. In order to find *R*_0_, then by the use of $V_{i}=V_{i}^{-}-V_{i}^{+}$ and the proposed model (1), yields that

$$\begin{array}{c} \frac{dx_{i}}{dt}=f_{i}\left(\chi \right)=F_{i}\left(\chi \right)-V_{i}\left(\chi \right), \end{array}$$ (7)

where,

$$\begin{array}{c} \begin{array}{cc} F_{i}\left(\chi \right) & =(\begin{array}{c} 0\\ 0\\ 0\\ \frac{\beta S(t)A(t)}{N}+\frac{\gamma \beta S(t)C(t)}{N}\\ 0\\ 0 \end{array}), \end{array} \end{array}$$ (8)

$$\begin{array}{c} \begin{array}{cc} V_{i}^{-}\left(\chi \right) & =(\begin{array}{c} \left(\sigma +d_{0}\right)L\left(t\right)\\ \left(d_{0}+\gamma_{1}\right)A\left(t\right)\\ \left(d_{0}+d_{1}+\gamma_{2}\right)C\left(t\right)\\ \frac{\beta S(t)A(t)}{N}+\frac{\gamma \beta S(t)C(t)}{N}+\left(d_{0}+\gamma_{3}\right)S\left(t\right)+b\xi NC\left(t\right)\\ d_{0}R\left(t\right)\\ bN\left(1-\xi \right)+\gamma_{3}S\left(t\right) \end{array}), \end{array} \end{array}$$ (9)

$$\begin{array}{c} \begin{array}{cc} V_{i}^{+}\left(\chi \right) & =(\begin{array}{c} 0\\ \sigma L(t)\\ b\xi \eta NC\left(t\right)+p\gamma_{1}A\left(t\right)\\ \gamma_{2}C\left(t\right)+\left(1-p\right)\gamma_{1}A\left(t\right)\\ b\xi N+\phi V(t)\\ bN\left(1-\xi \right)+\gamma_{3}S\left(t\right) \end{array}), \end{array} \end{array}$$ (10)

In Eqs (8)–(10), *F*_*i*_(*χ*), $V_{i}^{-}\left(\chi \right)$ and $V_{i}^{+}\left(\chi \right)$ represents the rate of appearance of new infections, the rate of transfer of individuals and the rate of transfer of individuals out of compartments, respectively. Since the hepatitis B virus transmission model (1) consist of non-negative initial conditions, thus we have the following conditions:

*A*_1_. if *χ* ≥ 0, then *F*_*i*_, $V_{i}^{-}$, $V_{i}^{+}\geq 0$ for *i* = 1…6.
*A*_2_. if *x*_*i*_ = 0, then $V_{i}^{-}=0$. In particular, if *x* ∈ *χ*_0_, then $V_{i}^{-}=0$ for *i* = 1…*m*.
*A*_3_. The incidence of infection for noninfected compartments is zero, i.e., *F*_*i*_ = 0, if *i* > *m*.
*A*_4_. if *x* ∈ *χ*_0_, then *F*_*i*_(*χ*) = 0 and $V_{i}^{+}=0$ for *i* = 1…*m*.
*A*_5_. if *F*_*i*_(*χ*) is set to zero, then all eigenvalues of $Df_{i}\left(\chi_{0}\right)=[\frac{\partial f_{i}}{\partial x_{j}}]$ have negative real parts.

Since the infected compartments are *L*, *A* and *C*, giving *m* = 3. An equilibrium solution with *L* = *A* = *C* = 0 has the form *F*_0_ = (*S*_0_, 0, 0, 0, 0, *V*_0_). Using *q*_2_ = *d*_0_ + *σ*, *q*_3_ = *d*_0_ + *γ*_1_, *q*_4_ = *d*_0_ + *d*_1_ + *γ*_2_ − *bξηN*, then without loss of generality, we obtain

$$\begin{array}{c} F=(\begin{array}{ccc} 0 & \frac{\beta S_{0}}{N} & \frac{\gamma \beta S_{0}}{N}\\ 0 & 0 & 0\\ 0 & 0 & 0 \end{array}),V=(\begin{array}{ccc} q_{2} & 0 & 0\\ -\sigma & q_{3} & 0\\ 0 & -p\gamma_{1} & q_{4} \end{array}), \end{array}$$ (11)

gives

$$\begin{array}{c} V^{-1}=(\begin{array}{ccc} \frac{1}{q_{2}} & 0 & 0\\ \frac{\sigma }{q_{2}q_{3}} & \frac{1}{q_{3}} & 0\\ \frac{\sigma p\gamma_{1}}{q_{2}q_{3}q_{4}} & \frac{p\gamma_{1}}{q_{3}q_{4}} & \frac{1}{q_{4}} \end{array}). \end{array}$$ (12)

The basic reproduction number, *R*_0_, is the spectral radius, *ρ*, of next generation matrix $\bar{K}=FV^{-1}$, i.e., *R*_0_ = *ρ*(*FV*^−1^) = max{|*λ*_1_|,…, |*λ*_3_|}. Thus the basic reproduction number, *R*_0_, for our proposed model (1) takes the form i.e., *R*_0_ = *γ*_01_+ *γ*_02_, where *γ*_01_ and *γ*_02_ is defined by the following equation:

$$\begin{array}{c} \gamma_{01}=\frac{\sigma \beta S_{0}}{Nq_{2}q_{3}},\;\gamma_{02}=\frac{\sigma \beta S_{0}\gamma \gamma_{1}p}{Nq_{2}q_{3}q_{4}},\;\text{and}\;S_{0}=\frac{bN(d_{0}+\phi )}{d_{0}\left(d_{0}+\phi +\gamma_{3}\right)}. \end{array}$$ (13)

### Sensitivity analysis of *R*_0_

Sensitivity analysis is recycled to define the relative significance of epidemic parameters to disease transmission and its dominance. It determines the robustness of model prediction to parameter values. Usually uncertainties in data collection and estimated values significantly affect the basic reproduction number.

**Definition 1**. *The normalized sensitivity index of the basic reproduction number R*_0_ *that depends differentiability on a parameter* Φ *is defined as*:

$$\begin{array}{c} S_{\Phi }=\frac{\Phi }{R_{0}}\frac{\partial R_{0}}{\partial \Phi }. \end{array}$$ (14)

We perform the analysis by calculating the sensitivity indices of the basic reproduction number to the parameters in the model. These indices allow us to measure the relative change in basic reproduction number with the change in a parameter. Using these indices, we find the parameters that highly affect the basic reproduction number and necessity to be targeted by control strategies.

**Fig 1:**
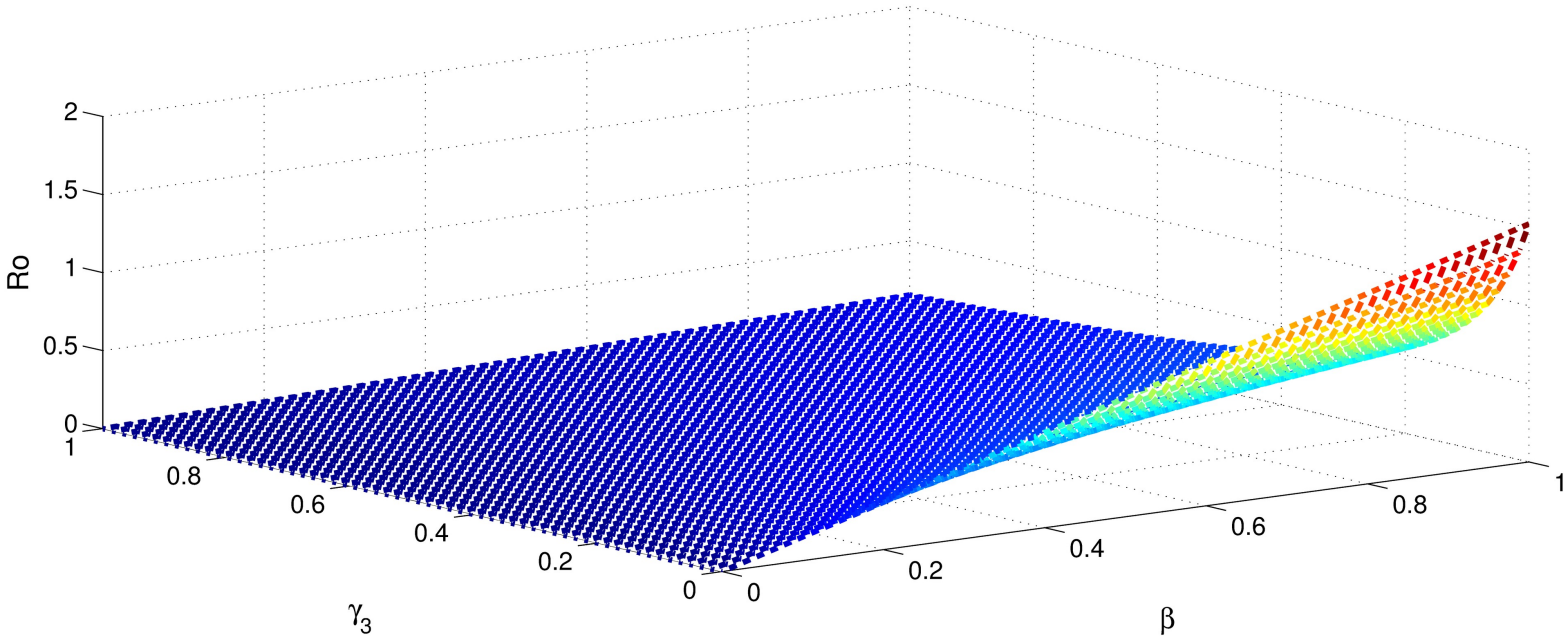
The sensitivity analysis of the basic reproduction number *R*_0_ verses *β* and *γ*_3_.

**Fig 2:**
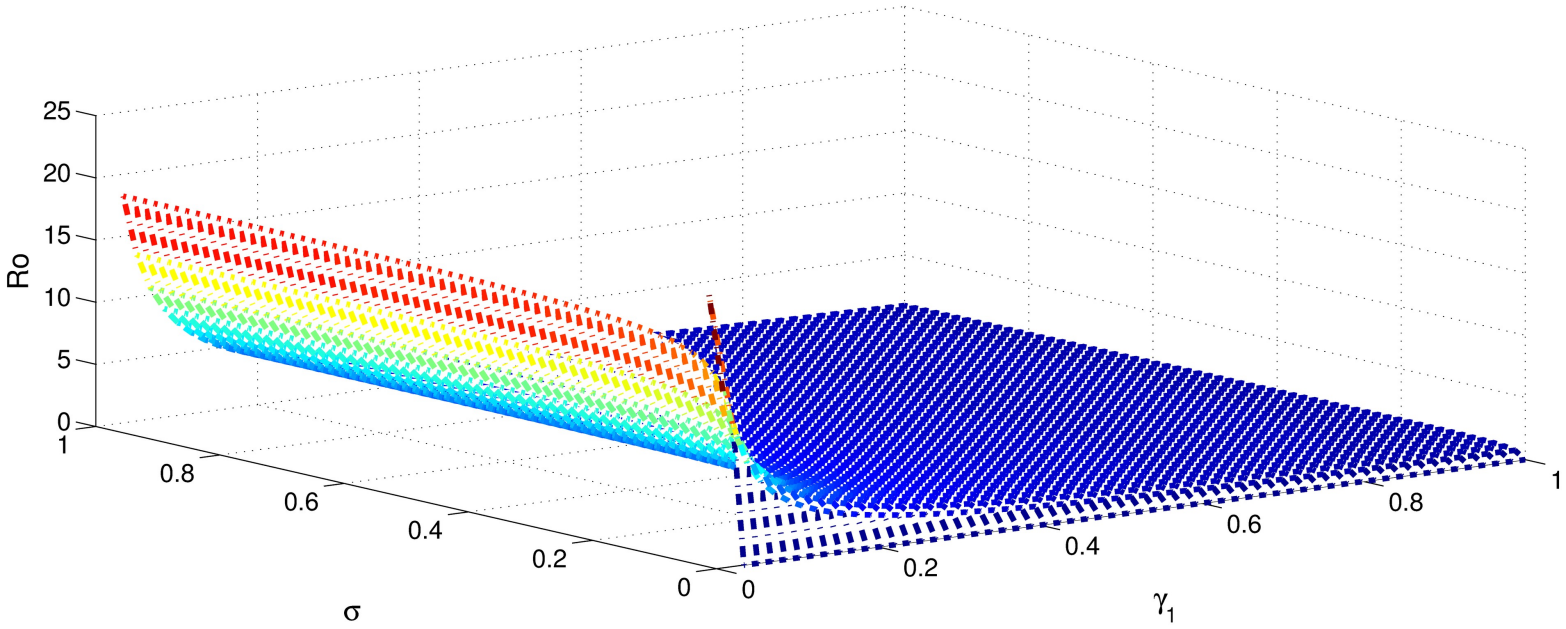
The sensitivity analysis of the basic reproduction number *R*_0_ verses *γ*_1_ and *σ*.

**Fig 3:**
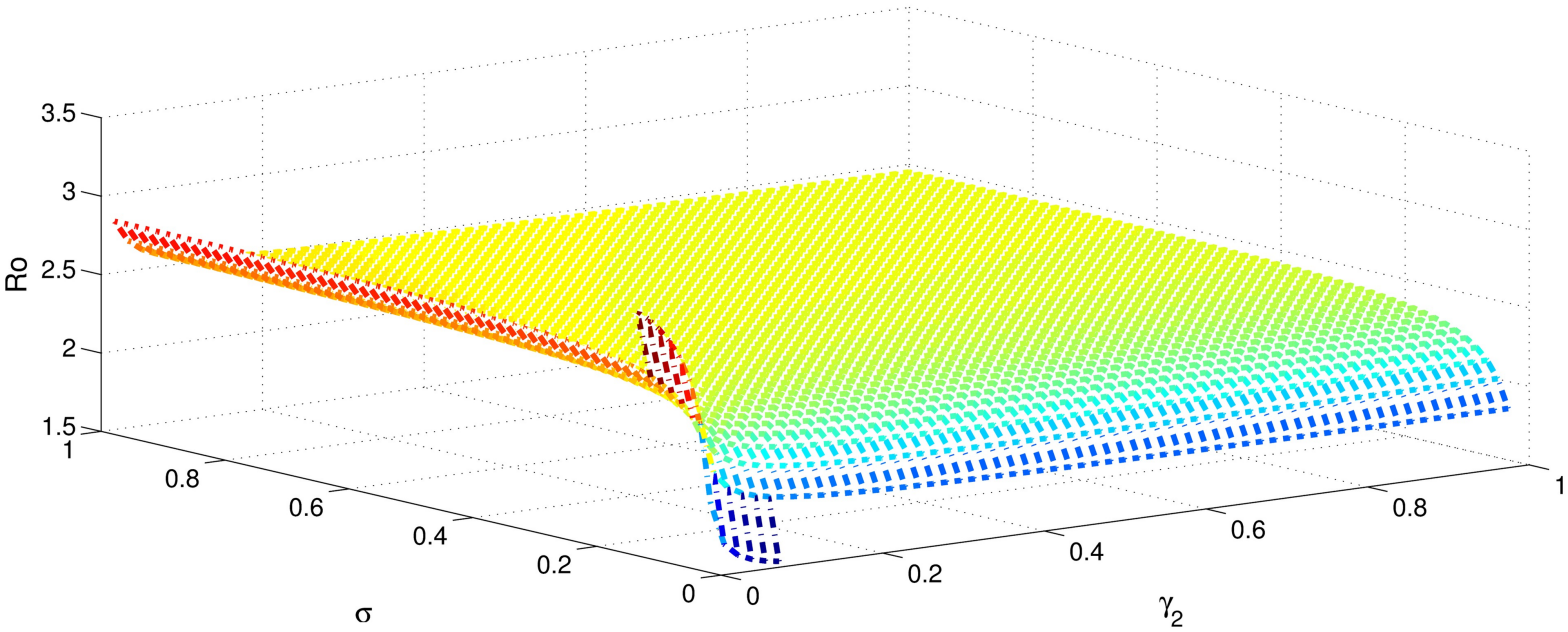
The sensitivity analysis of the basic reproduction number *R*_0_ verses *γ*_2_ and *σ*.

**Fig 4:**
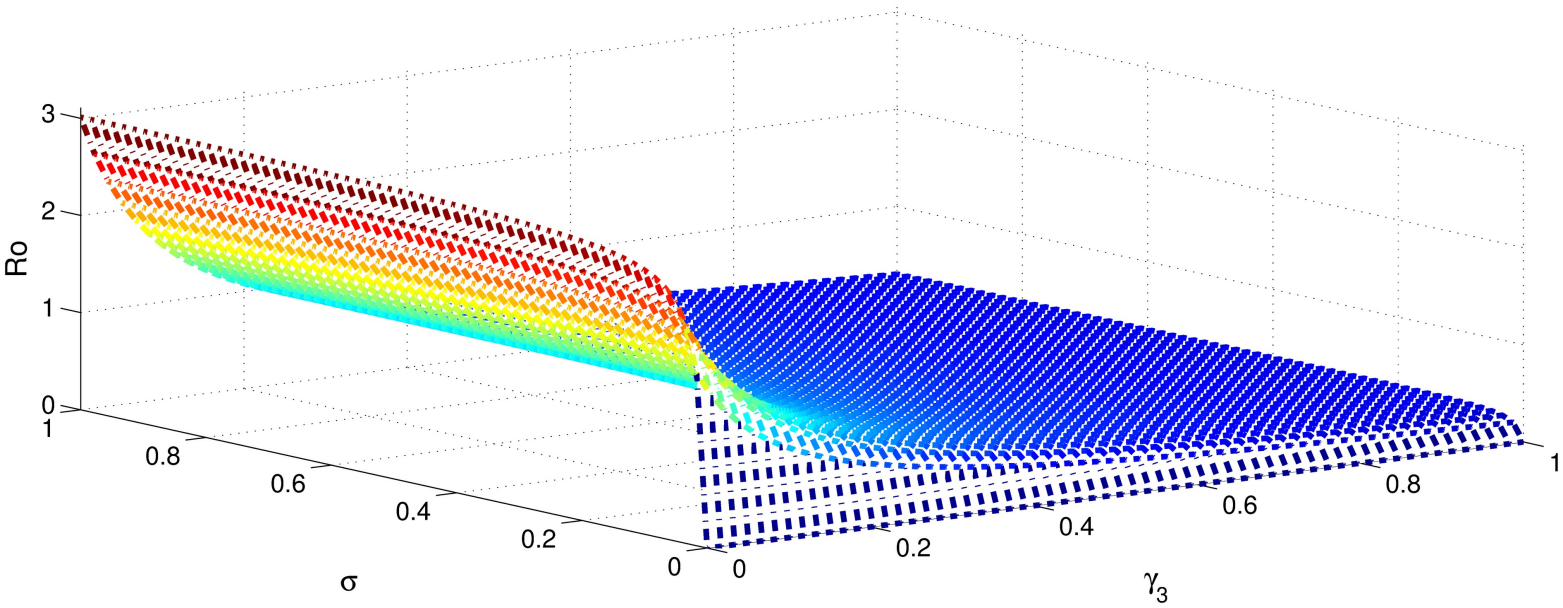
The sensitivity analysis of the basic reproduction number *R*_0_ verses *γ*_3_ and *σ*.

**Fig 5:**
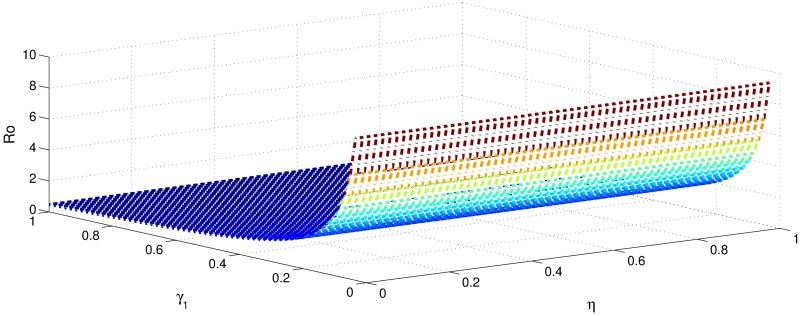
The sensitivity analysis of the basic reproduction number *R*_0_ verses *η* and *γ*_1_.

**Fig 6:**
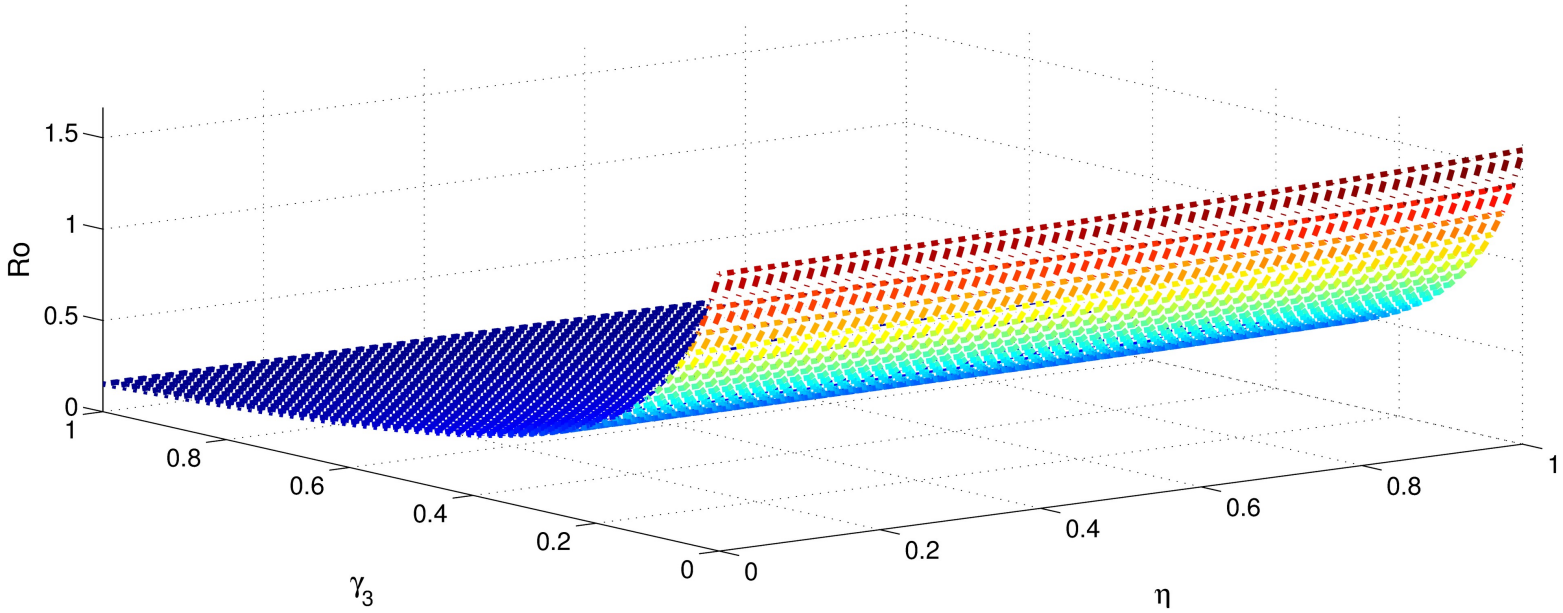
The sensitivity analysis of the basic reproduction number *R*_0_ verses *η* and *γ*_3_.

**Fig 7:**
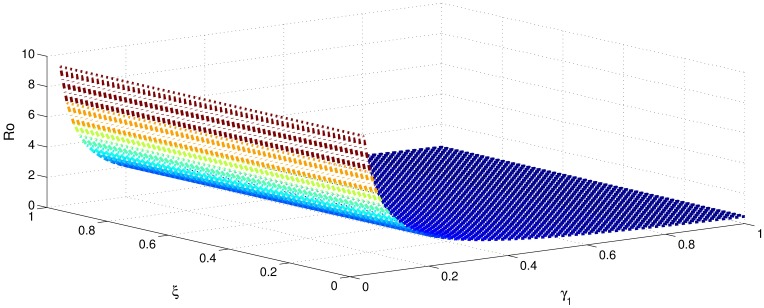
The sensitivity analysis of the basic reproduction number *R*_0_ verses *γ*_1_ and *ξ*.

**Fig 8:**
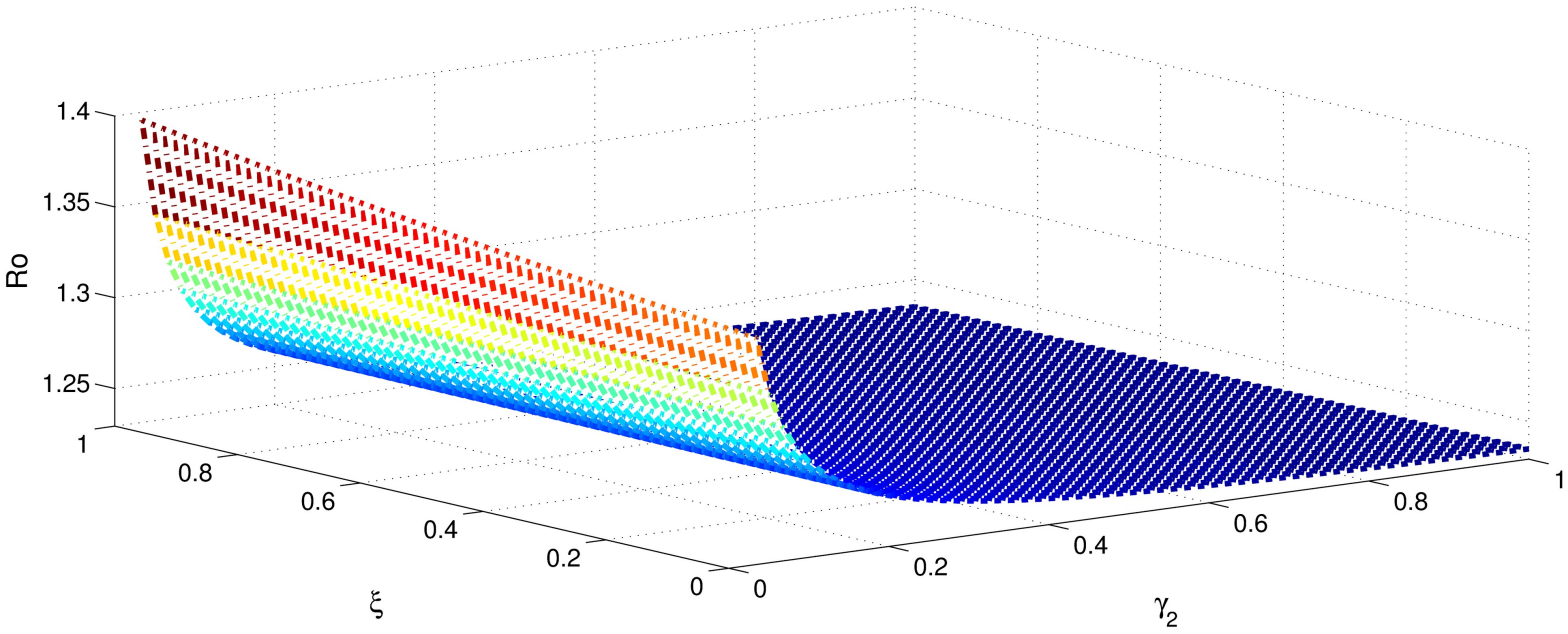
The sensitivity analysis of the basic reproduction number *R*_0_ verses *γ*_2_ and *ξ*.

**Fig 9:**
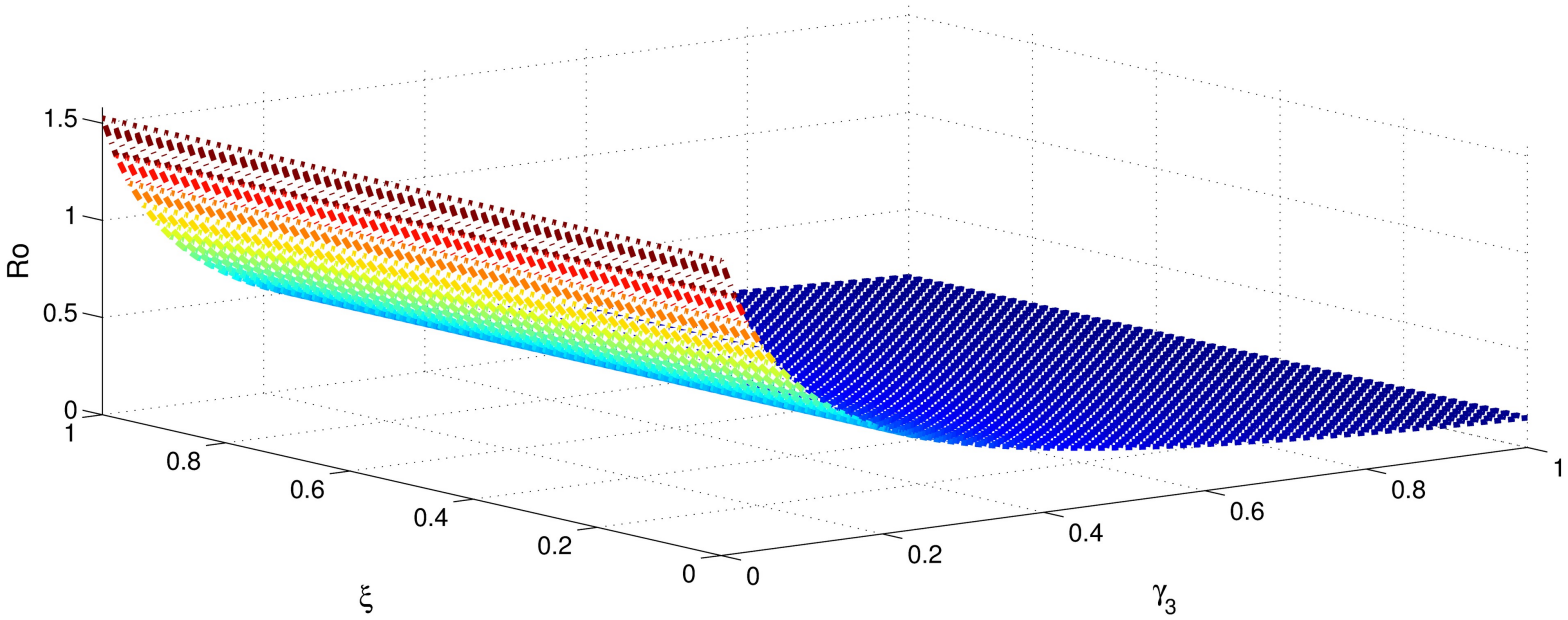
The sensitivity analysis of the basic reproduction number *R*_0_ verses *γ*_3_ and *ξ*.

**Fig 10:**
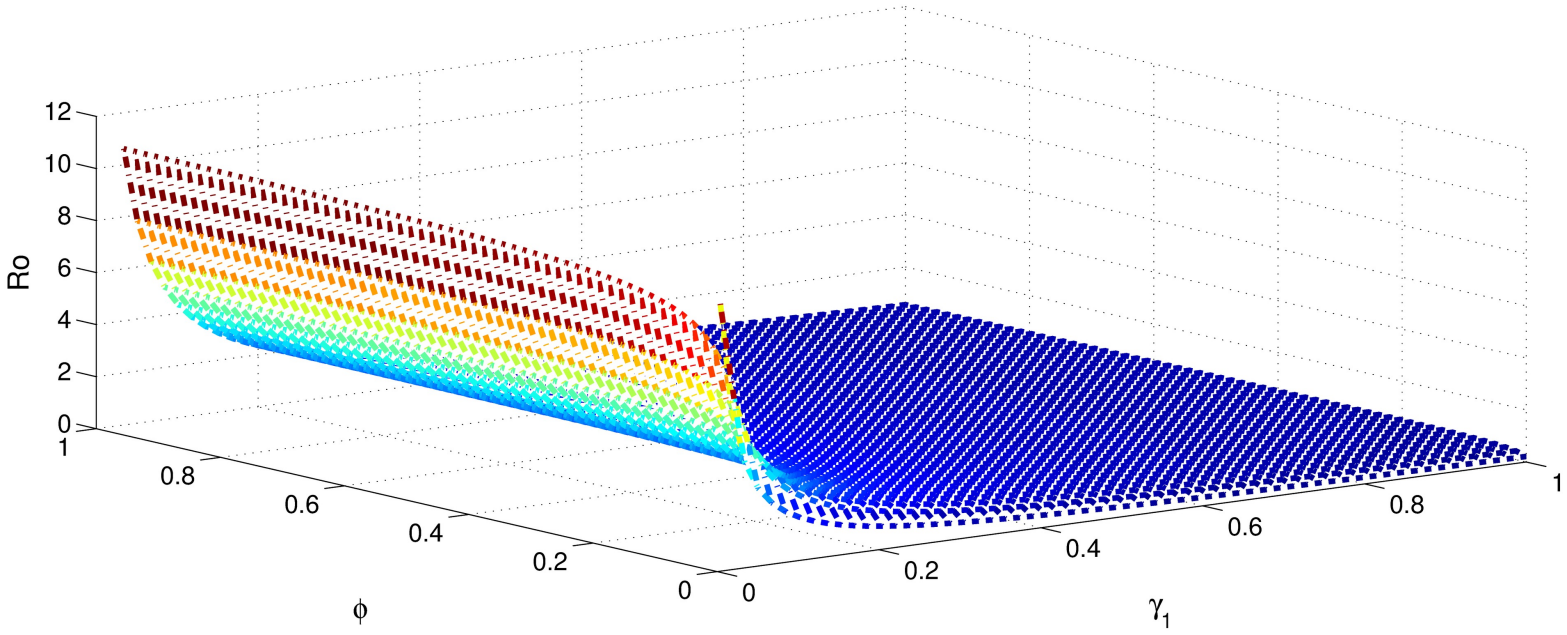
The sensitivity analysis of the basic reproduction number *R*_0_ verses *γ*_1_ and *ϕ*.

**Fig 11:**
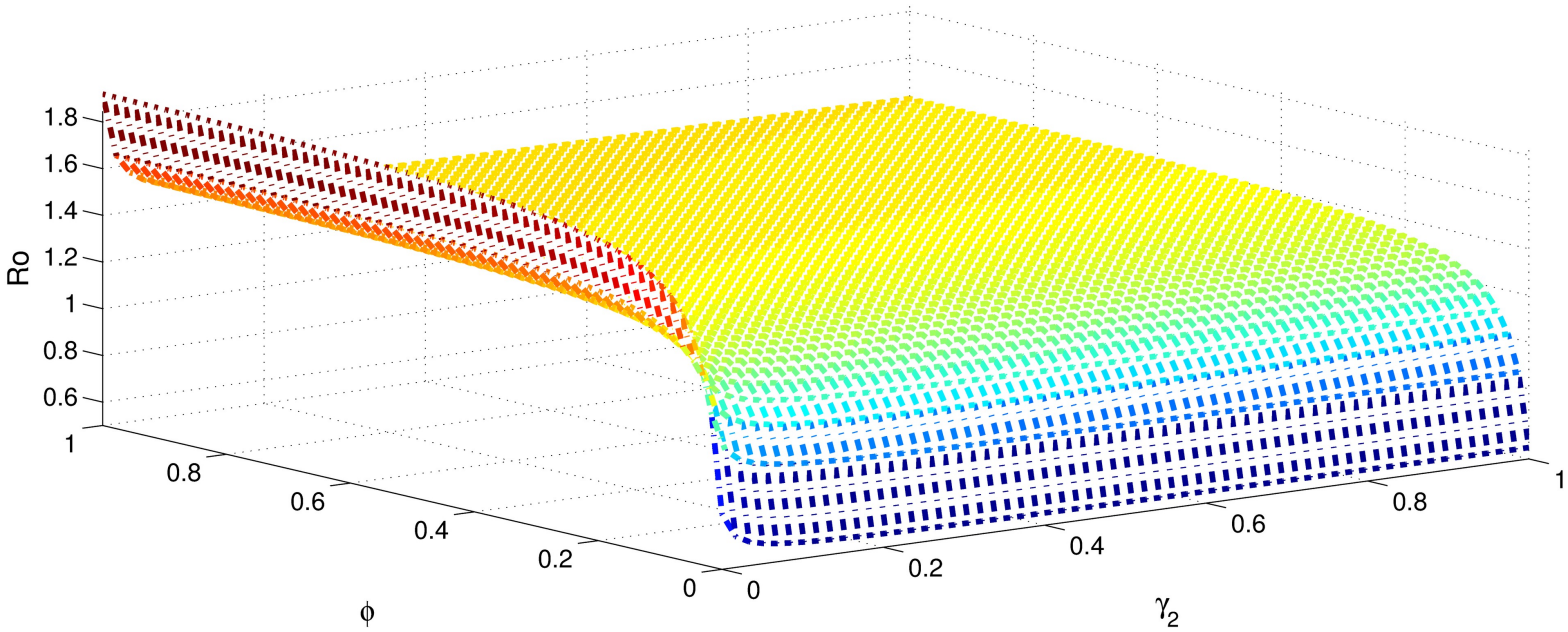
The sensitivity analysis of the basic reproduction number *R*_0_ verses *γ*_2_ and *ϕ*.

**Fig 12:**
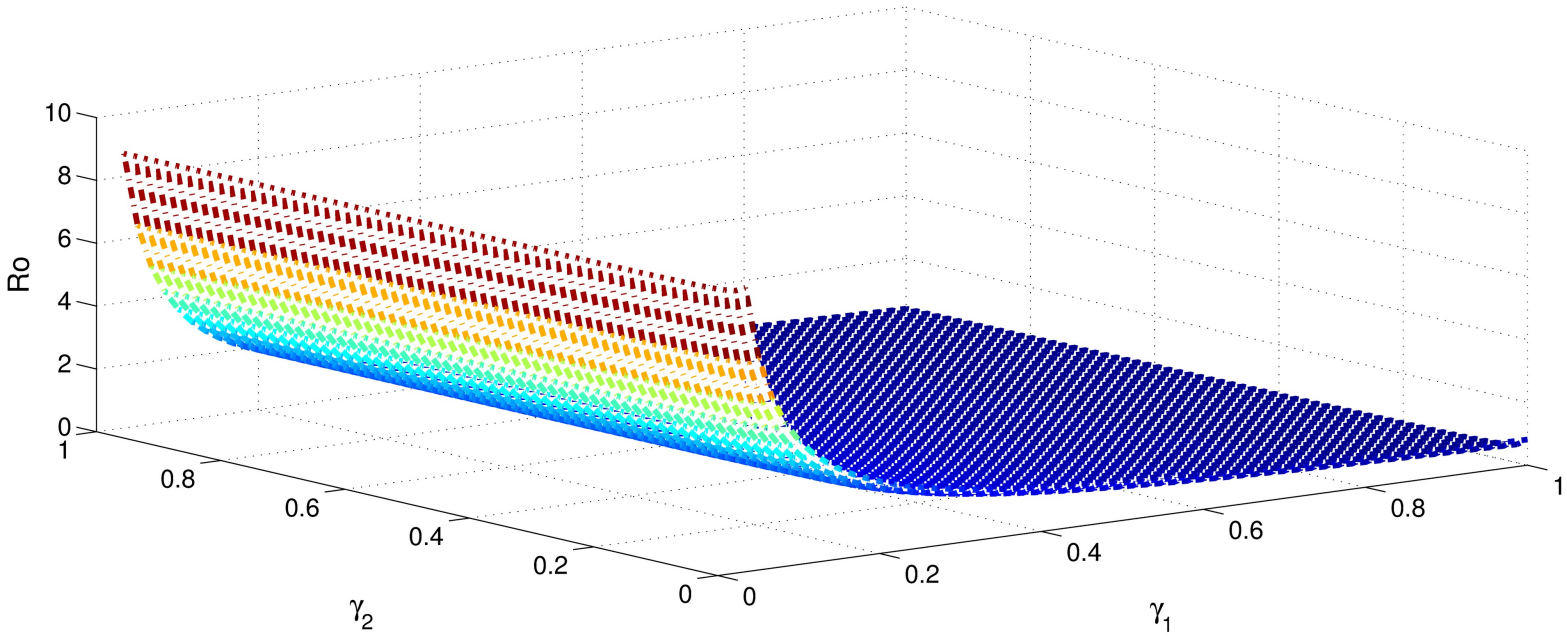
The sensitivity analysis of the basic reproduction number *R*_0_ verses *γ*_1_ and *γ*_2_.

Table 1 shows that the parameters *β*, *σ*, *ξ*, *η* and *ϕ* have a positive influence in the rate of basic reproduction number. This describes that the growth or decay of these parameters say by 10 percent, then the basic reproduction number will increase or decrease by 10 percent, 9.0 percent, 5.46 percent, 5.46 percent and 8.13 percent, respectively as shown in Figs 1–6. But the index for parameters *γ*_1_ and *γ*_3_ illustrate, that increasing their values by 10 percent will decrease the values of basic reproduction number *R*_0_ by 8.24 percent, 7.38 percent and 1.5 percent, respectively shown in Figs 7–12.

**Table 1:** Sensitivity indices of *R*_0_ with respect to some chosen parameters.

Parameter	Sensitivity index	value
Hepatitis B transmission rate (*β*)	*S*_*β*_	+1.0000
Moving rate from L to A (*σ*)	*S*_*σ*_	+0.9097
Recovery rate in A (*γ*_1_)	$S_{\gamma_{1}}$	-0.8247
Recovery rate in C (*γ*_2_)	$S_{\gamma_{2}}$	-0.7385
Vaccination (*γ*_3_)	$S_{\gamma_{3}}$	-0.1514
Proportion of parentally infected individuals (*η*)	*S*_*η*_	+0.5460
Birth rate without successful vaccination (*ξ*)	*S*_*ξ*_	+0.5460
waning vaccine induced immunity rate (*ϕ*)	*S*_*ϕ*_	+0.8135

In order to control the infection of hepatitis B, we focus to control the transmission of hepatitis B infection *β*, which has got highest sensitivity index 1. This means that decrease in transmission rate by 10 percent would decrease basic reproduction by 10 percent. The second highest sensitivity index is −0.8247 is that of recovery rate *γ*_1_. That is increasing *γ*_1_ by 10 percent will decrease basic reproduction number by 8.24 percent. The parameters *σ*, *η*, *ξ* and *ϕ* collectively have got the sensitivity index 2.8152. So decreasing these parameters by 10 percent causes collectively decreases basic reproduction number by 28.152 percent. Similarly the parameters *γ*_1_ and *γ*_2_, have got the sensitivity index 1.5623. So increasing the treatment of hepatitis B infected individuals (acutely and chronically) will decrease the basic reproduction number by 15.632 percent. Therefore, it is easy to develop a control strategy.

## Steady state analysis

In order to study the dynamic of the model (1), we can reduce the proposed model by eliminating *R*(*t*); because *R*(*t*) appears only in the fifth equation of the proposed model, therefore we can discuss the following reduced system:

$$\begin{array}{c} \begin{array}{cc} \frac{dS(t)}{dt} & =b\xi N\left(1-\eta C\left(t\right)\right)+\phi V\left(t\right)-\frac{\beta S(t)A(t)}{N}-\frac{\gamma \beta S(t)C(t)}{N}-\left(d_{0}+\gamma_{3}\right)S\left(t\right),\\ \frac{dL(t)}{dt} & =\frac{\beta S(t)A(t)}{N}+\frac{\gamma \beta S(t)C(t)}{N}-\left(d_{0}+\sigma \right)L\left(t\right),\\ \frac{dA(t)}{dt} & =\sigma L\left(t\right)-\left(d_{0}+\gamma_{1}\right)A\left(t\right),\\ \frac{dC(t)}{dt} & =b\xi \eta NC\left(t\right)+p\gamma_{1}A\left(t\right)-\left(d_{0}+d_{1}+\gamma_{2}\right)C\left(t\right),\\ \frac{dV(t)}{dt} & =bN\left(1-\xi \right)+\gamma_{3}S\left(t\right)-\left(d_{0}+\phi \right)V\left(t\right). \end{array} \end{array}$$ (15)

The reduced model (15) has a disease free equilibrium, which is denoted by *F*_0_ and define as *F*_0_ = (*S*_0_, 0, 0, 0, 0, *V*_0_), where

$$\begin{array}{c} S_{0}=\frac{Nb(\phi +\xi d_{0})}{d_{0}\left(d_{0}+\phi +\gamma_{3}\right)},\;V_{0}=\frac{Nb(d_{0}-d_{0}\xi +\gamma_{3})}{d_{0}\left(d_{0}+\phi +\gamma_{3}\right)}. \end{array}$$ (16)

Similarly, the unique positive disease endemic state of the model (15) is denoted by F_*_ and define as F_*_ = (S_*_, L_*_, A_*_, C_*_, R_*_, V_*_), which exist only if *R*_0_ > 1. Using the values of *q*_*i*_ for *i* = 1, 2, 3…5 are as *q*_1_ = *d*_0_ + *γ*_3_, *q*_2_ = *d*_0_ + *σ*, *q*_3_ = *d*_0_ + *γ*_1_, *q*_4_ = *d*_0_ + *d*_1_ + *γ*_2_ − *bξηN* and *q*_5_ = *d*_0_ + *ϕ*, the components of the disease endemic equilibrium F_*_ takes the following form

$$\begin{array}{c} \begin{array}{cc} S_{*} & =\frac{q_{2}q_{3}q_{4}}{q_{4}+p\gamma \gamma_{1}},\;L_{*}=\frac{d_{0}q_{2}q_{3}^{2}q_{4}\left(\gamma_{3}+q_{5}\right)\left(q_{4}-b\xi \eta N\right)(R_{0}-1)}{\sigma q_{5}\left(p\gamma \gamma_{1}+q_{4}\right)\left(\left(q_{4}+p\gamma \gamma_{1}\right)\beta S_{*}+b\xi \eta p\gamma_{1}\right)},\\ A_{*} & =\frac{d_{0}q_{2}q_{3}q_{4}^{2}\left(\gamma_{3}+q_{5}\right)\left(R_{0}-1\right)}{q_{5}\left(p\gamma \gamma_{1}+q_{4}\right)\left(\left(q_{4}+p\gamma \gamma_{1}\right)\beta S_{*}+b\xi \eta Np\gamma_{1}\right)},\\ C_{*} & =\frac{d_{0}\gamma \beta p\gamma_{1}q_{3}\left(\gamma_{3}+q_{5}\right)S_{*}^{2}(R_{0}-1)}{q_{5}\left(\left(q_{4}+p\gamma \gamma_{1}\right)\beta S_{*}+b\xi \eta Np\gamma_{1}\right)},\;V_{*}=\frac{1}{q_{5}}\left(bN\left(1-\xi \right)+\gamma_{3}S_{*}\right). \end{array} \end{array}$$ (17)

Thus, we conclude that there is no disease endemic equilibrium, whenever *R*_0_ < 1 and a unique positive (or endemic) equilibrium, otherwise.

### Stability analysis of DFE

To investigate the stability analysis of the disease free equilibrium point *F*_0_, we make use of the following results.

**Theorem 1**. *If R*_0_ < 1, *then the disease free equilibrium point F*_0_ = (*S*_0_, 0, 0, 0, 0, *V*_0_) *is locally asymptotically stable and if R*_0_ > 1 *then it is unstable*.

**Proof**: The Jacobian matrix of the model (15) at disease free equilibrium point *F*_0_ becomes

$$\begin{array}{c} J\left(F_{0}\right)=(\begin{array}{ccccc} -q_{1} & 0 & -\frac{\beta S_{0}}{N} & -(b\xi \eta N+\frac{\gamma \beta S_{0}}{N}) & \phi \\ 0 & -q_{2} & \frac{\beta S_{0}}{N} & \frac{\gamma \beta S_{0}}{N} & 0\\ 0 & \sigma & -q_{3} & 0 & 0\\ 0 & 0 & p\gamma_{1} & -q_{4} & 0\\ \gamma_{3} & 0 & 0 & 0 & -q_{5} \end{array}). \end{array}$$ (18)

Using *b*_*i*_ for *i* = 1, 2, 3, such that *b*_0_ = *q*_2_*q*_3_*q*_4_(1 − *R*_0_), *b*_1_ = *q*_4_(2*d*_0_ + *σ* + *γ*_1_) + *q*_2_*q*_3_(1 − *γ*_01_) and *b*_2_ = 2*d*_0_ + *σ* + *q*_4_. The characteristic equation of the Jacobian matrix (18) has the form

$$\begin{array}{c} \left(\lambda +d_{0}\right)\left(\lambda +d_{0}+\gamma_{3}+\phi \right)\left(\lambda^{3}+b_{2}\lambda^{2}+b_{1}\lambda +b_{0}\right)=0. \end{array}$$ (19)

The fundamental theorem of algebra reveals that there are five roots of Eq (19). Hence, the Jacobian matrix *J*(*F*_0_) (18) has five eigenvalues. Clearly for *R*_0_ < 1, two eigenvalues of them are *λ*_1_ = −*d*_0_ and *λ*_2_ = −*d*_0_ − *γ*_3_ − *ϕ* among them has negative real parts. The remaining three eigenvalues are obtained by solving

$$\begin{array}{c} p\left(\lambda \right)=\lambda^{3}+b_{2}\lambda^{2}+b_{1}\lambda +b_{0}. \end{array}$$ (20)

Roots of Eq (20) have negative real parts, if the Routh-Hurwitz criterion (*H*_1_): *b*_1_ > 0, *b*_0_ > 0 and *b*_1_*b*_2_ > *b*_0_ holds, which implies that

$$\begin{array}{c} b_{1}b_{2}-b_{0}=\left(2d_{0}+\sigma +\gamma_{1}\right)\left(1-\gamma_{01}\right)+q_{4}\left(2d_{0}+\sigma +\gamma_{1}\right)\left(2d_{0}+\sigma +\gamma_{1}+q_{4}\right)+\sigma \beta S_{0}p\gamma \gamma_{1}>0. \end{array}$$ (21)

Thus, it can be noted that (*H*_1_) holds if and only if *R*_0_ < 1. Therefore, by the Routh-Hurwitz criterion, all the eigenvalues have negative real parts, so *F*_0_ is locally asymptotically stable.

**Theorem 2**. *If R*_0_ < 1, *the disease free equilibrium point F*_0_ *is globally asymptotically stable and unstable, if R*_0_ > 1.

**Proof**: Let us construct the Lyapunov function

$$\begin{array}{ll} \Gamma \left(t\right) & =\frac{1}{2}{\left[\left(S-S_{0}\right)+L\left(t\right)+A\left(t\right)+C\left(t\right)+R\left(t\right)+\left(V-V_{0}\right)\right]}^{2}+k_{1}\left(S-S_{0}\right)+k_{2}L\left(t\right)\\ & +k_{3}A\left(t\right)+k_{4}C\left(t\right)+k_{5}\left(V-V_{0}\right), \end{array}$$ (22)

where *k*_*i*_ for *i* = 1, 2, 3, 4, 5 are positive constants to be determined. Differentiating Eq (22) with respect to *t* and using the system (1), we obtain

$$\begin{array}{c} \begin{array}{cc} \frac{d\Gamma (t)}{dt} & =(\left(S-S_{0}\right)+L\left(t\right)+A\left(t\right)+C\left(t\right)+R\left(t\right)+\left(V-V_{0}\right))\left(bN-d_{0}N\left(t\right)-d_{1}C\left(t\right)\right)\\ & +k_{1}(b\xi N\left(1-\eta C\left(t\right)\right)+\phi V\left(t\right)-\frac{\beta S(t)A(t)}{N}-\frac{\gamma \beta S(t)C(t)}{N}-\left(d_{0}+\gamma_{3}\right)S\left(t\right))\\ & +k_{2}(\frac{\beta S(t)A(t)}{N}+\frac{\gamma \beta S(t)C(t)}{N}-\left(d_{0}+\sigma \right)L\left(t\right))+k_{3}(\sigma L\left(t\right)-\left(d_{0}+\gamma_{1}\right)A\left(t\right))\\ & +k_{4}(b\xi \eta NC\left(t\right)+p\gamma_{1}A\left(t\right)-\left(d_{0}+d_{1}+\gamma_{2}\right)C\left(t\right))\\ & +k_{5}(\gamma_{2}C\left(t\right)+\left(1-p\right)\gamma_{1}A\left(t\right)-d_{0}R\left(t\right))+k_{6}(bN\left(1-\xi \right)+\gamma_{3}S\left(t\right)-\left(d_{0}+\phi \right)V\left(t\right)). \end{array} \end{array}$$ (23)

By choosing the positive constants *k*_*i*_ = (*d*_0_ + *σ*)(*d*_0_ + *d*_1_ + *γ*_2_ − *bξηN*) for *i* = 1, 2, 3, 5 and *k*_4_ = *σβγS*_0_, then Eq (23) reduce to the following equation

$$\begin{array}{c} \begin{array}{cc} \frac{d\Gamma (t)}{dt} & =-{\left(\left(S-S_{0}\right)+L\left(t\right)+A\left(t\right)+C\left(t\right)+R\left(t\right)+\left(V-V_{0}\right)\right)}^{2}\\ & -(\left(S-S_{0}\right)+L\left(t\right)+A\left(t\right)+C\left(t\right)+R\left(t\right)+\left(V-V_{0}\right))d_{1}C\left(t\right)\\ & -b\xi \eta N\left(\sigma +d_{0}\right)\left(d_{0}+d_{1}+\gamma_{2}-b\xi \eta N\right)C\left(t\right)\\ & -d_{0}\left(\sigma +d_{0}\right)\left(d_{0}+d_{1}+\gamma_{2}-b\xi \eta N\right)L\left(t\right)\\ & -\left(\sigma +d_{0}\right)\left(\gamma_{1}+d_{0}\right)\left(d_{0}+d_{1}+\gamma_{2}-b\xi \eta N\right)\left(1-\gamma_{02}\right)\\ & -\sigma \beta \gamma S_{0}\left(d_{0}+d_{1}+\gamma_{2}-b\xi \eta N\right)C\left(t\right)\\ & -\left(\sigma +d_{0}\right)\left(d_{0}+d_{1}+\gamma_{2}-b\xi \eta N\right)\left(d_{0}\left(S-S_{0}\right)+\left(V-V_{0}\right)\right). \end{array} \end{array}$$ (24)

Eq (24) showing that, if *R*_0_ < 1, we have 0 < *γ*_02_ < 1, therefore $\frac{d\Gamma (t)}{dt}$ is negative. Also $\frac{d\Gamma (t)}{dt}=0,$ if *S* = *S*_0_, *L* = *L*_0_, *A* = *A*_0_, *C* = *C*_0_, *R* = *R*_0_ and *V* = *V*_0_, thus the largest compact invariant set in Ω is the singleton set {*F*_0_}, so LaSalle’s invariant principle implies that, the disease free equilibrium point *F*_0_ is globally asymptotically stable.

### Stability analysis of EE

To investigate the stability analysis of the endemic equilibrium point F_*_, we prove the following results.

**Theorem 3**. *If R*_0_ > 1, then the endemic equilibrium point F_*_ = (S_*_, L_*_, A_*_, C_*_, R_*_, V_*_) *is locally asymptotically stable and if R*_0_ < 1 *then it is unstable*.

**Proof**: Using the elementary row transformation for the Jacobian matrix of the model (15) around F_*_, we obtain the following matrix

$$\begin{array}{c} J\left(F_{*}\right)=(\begin{array}{ccccc} -q_{1}-\frac{\beta S_{*}}{N}-\frac{\gamma \beta S_{*}}{N} & 0 & -\frac{\beta S_{*}}{N} & -(b\xi \eta N+\frac{\gamma \beta S_{*}}{N}) & \phi \\ 0 & -q_{2} & H_{1} & \gamma H_{1}-b\xi \eta H_{2} & \phi H_{2}\\ 0 & 0 & -q_{3}+\frac{\sigma H_{1}}{q_{2}} & \frac{\sigma (\gamma H_{1}-b\xi \eta H_{2})}{q_{2}} & \frac{\sigma \phi H_{2}}{q_{2}}\\ 0 & 0 & 0 & H_{3}-b\xi \eta NH_{4} & \phi H_{4}\\ 0 & 0 & 0 & 0 & H_{5} \end{array}), \end{array}$$ (25)

where

$$\begin{array}{c} \begin{array}{cc} H_{1} & =\frac{\beta S_{*}q_{1}}{d_{0}N+\beta A_{*}+\gamma \beta C_{*}+\gamma_{3}N},\;H_{2}=\frac{\beta A_{*}+\gamma \beta^{2}C_{*}}{d_{0}N+\beta A_{*}+\gamma \beta C_{*}+\gamma_{3}N},\\ H_{3} & =-q_{4}-\frac{q_{1}\beta p\gamma \gamma_{1}S_{*}}{\sigma \beta q_{1}S_{*}-q_{2}q_{3}\left(d_{0}N+\beta A_{*}+\gamma \beta C_{*}+\gamma_{3}N\right)},\\ H_{4} & =\frac{\sigma \beta p\gamma_{1}\left(A_{*}+\gamma C_{*}\right)}{q_{2}q_{3}\left(d_{0}N+\beta A_{*}+\gamma \beta C_{*}+\gamma_{3}N\right)-q_{1}\sigma \beta S_{*}},\\ H_{5} & =\frac{\gamma_{3}\phi H_{4}(p\gamma_{1}\left(b\xi \eta N^{2}+\gamma \beta S_{*}\right)+q_{4}\beta S_{*})}{p\gamma_{1}\left(d_{0}N+\beta A_{*}+\gamma \beta C_{*}+\gamma_{3}N\right)\left(H_{3}-b\xi \eta NH_{4}\right)}-\frac{q_{5}\left(d_{1}N+\beta A_{*}+\gamma \beta C_{*}\right)+d_{0}\gamma_{3}N}{d_{0}N+\beta A_{*}+\gamma \beta C_{*}+\gamma_{3}N}. \end{array} \end{array}$$ (26)

The eigenvalues of the Jacobian matrix around the disease endemic equilibrium F_*_ are

$$\begin{array}{c} \begin{array}{cc} \lambda_{1} & =-\frac{1}{N}\left(Nq_{1}+\beta A_{*}+\gamma \beta C_{*}\right),\;\lambda_{2}=-q_{2},\\ \lambda_{3} & =-\frac{q_{2}q_{3}\left(d_{0}N+\beta A_{*}+\gamma \beta C_{*}+\gamma_{3}N\right)-\sigma q_{1}\beta S_{*}}{q_{2}\left(d_{0}N+\beta A_{*}+\gamma \beta C_{*}+\gamma_{3}N\right)},\\ \lambda_{4} & =H_{3}-b\xi \eta NH_{4},\;\lambda_{5}=H_{5}. \end{array} \end{array}$$ (27)

Clearly two eigenvalues of matrix (25) have negative real parts, i.e. *λ*_1_ < 0 and *λ*_2_ < 0, while *λ*_3_, *λ*_4_ and *λ*_5_ have negative real parts if and only if

$$\begin{array}{c} \begin{array}{cc} \frac{\beta p\gamma \gamma_{1}S_{*}}{q_{2}q_{4}\left(d_{0}N+\beta A_{*}+\gamma \beta C_{*}+\gamma_{3}N\right)-\gamma \beta S_{*}q_{4}} & <1,\;\frac{\sigma q_{1}\beta S_{*}}{q_{2}q_{3}\left(d_{0}N+\beta A_{*}+\gamma \beta C_{*}+\gamma_{3}N\right)}<1, \end{array} \end{array}$$ (28)

which holds as F_*_ exist. Therefore, all eigenvalues contains negative real parts, and we have the conclusion, that the endemic (disease) equilibrium point F_*_ is locally asymptotically stable.

**Theorem 4**. *If R*_0_ > 1, then the endemic equilibrium point F_*_ = (S_*_, L_*_, A_*_, C_*_, R_*_, V_*_) *is globally asymptotically stable and unstable otherwise*.

**Proof**: Let *J* and *J*^∣2∣^ be the Jacobian matrices and second additive compound matrix of the system containing only the first three equation of the model (1), which becomes

$$J=(\begin{array}{ccc} -a_{11} & 0 & -a_{13}\\ a_{21} & a_{22} & a_{23}\\ 0 & \sigma & -a_{33} \end{array}),\;\;J^{|2|}=(\begin{array}{ccc} -(a_{11}+a_{22}) & a_{23} & -a_{13}\\ a_{32} & -(a_{11}+a_{33}) & a_{12}\\ -a_{31} & a_{21} & -(a_{22}+a_{33}) \end{array}).$$

Let us consider the function $P\left(\chi \right)=P\left(S,L,A\right)=diag\{\frac{S}{L},\frac{S}{L},\frac{S}{L}\},$ which implies that $P^{-1}\left(\chi \right)=diag\{\frac{L}{S},\frac{L}{S},\frac{L}{S}\},$ then taking the time derivative, that is *P*_*f*_(*χ*), we get

$$\begin{array}{c} P_{f}\left(\chi \right)=diag\{\frac{\dot{S}}{S}-\frac{S\dot{L}}{L^{2}},\frac{\dot{S}}{S}-\frac{S\dot{L}}{L^{2}},\frac{\dot{S}}{S}-\frac{S\dot{L}}{L^{2}}\}. \end{array}$$ (29)

Now $P_{f}P^{-1}=diag\{\frac{\dot{S}}{S}-\frac{\dot{L}}{L},\frac{\dot{S}}{S}-\frac{\dot{L}}{L},\frac{\dot{S}}{S}-\frac{\dot{L}}{L}\}$ and $PJ_{2}^{|2|}P^{-1}=J_{2}^{|2|}$. Thus we take $B=P_{f}P^{-1}+PJ_{2}^{|2|}P^{-1}$, which can be written as

$$\begin{array}{c} B=(\begin{array}{cc} B_{11} & B_{12}\\ B_{21} & B_{22} \end{array}), \end{array}$$ (30)

where

$$\begin{array}{cc} B_{11} & =\frac{\dot{S}}{S}-\frac{\dot{L}}{L}-\frac{1}{N}\beta A-\frac{1}{N}\gamma \beta B-2d_{0}-\gamma_{3}-\sigma ,\\ B_{12} & =[\begin{array}{cc} \frac{1}{N}\beta S & \frac{1}{N}\beta S \end{array}],\;B_{21}=[\begin{array}{c} \sigma \\ 0 \end{array}], \end{array}$$

$$\begin{array}{c} B_{22}=[\begin{array}{cc} \frac{\dot{S}}{S}-\frac{\dot{L}}{L}-\frac{1}{N}\beta A-\frac{1}{N}\gamma \beta B-2d_{0}-\gamma_{1}-\sigma & 0\\ \frac{1}{N}\beta A+\frac{1}{N}\gamma \beta B & \frac{\dot{S}}{S}-\frac{\dot{L}}{L}-2d_{0}-\gamma_{1}-\sigma \end{array}]. \end{array}$$

Let (*b*_1_, *b*_2_, *b*_3_) be a vector in *R*^3^ and its norm ‖.‖ defined by

$$\begin{array}{c} ∥b_{1},b_{2},b_{3}∥=max\{∥b_{1}∥,∥b_{2}∥+∥b_{3}∥\}. \end{array}$$ (31)

Now we take the Lozinski measure *ℓ*(*B*) with respect to the above norm described by Martin et. al. in 1974 [23], that is *ℓ*(*B*) ≤ *sup*{*g*_1_, *g*_2_} = *sup*{*ℓ*(*B*_11_) + ‖*B*_12_)‖, *ℓ*(*B*_22_) + ‖*B*_21_‖}, where *g*_*i*_ = *ℓ*(*B*_*ii*_) + ‖*B*_*ij*_)‖ for *i* = 1, 2 and *i* ≠ *j*, which implies that

$$\begin{array}{c} g_{1}=\ell \left(B_{11}\right)+∥B_{12})∥,\;\;g_{2}=\ell \left(B_{22}\right)+∥B_{21})∥, \end{array}$$ (32)

where $\ell \left(B_{11}\right)=\frac{\dot{S}}{S}-\frac{\dot{L}}{L}-\frac{1}{N}\beta A-\frac{1}{N}\gamma \beta B-2d_{0}-\gamma_{3}-\sigma$, $\ell \left(B_{22}\right)=max\{\frac{\dot{S}}{S}-\frac{\dot{L}}{L}-2d_{0}-\gamma_{3}-\gamma_{1},\frac{\dot{S}}{S}-\frac{\dot{L}}{L}-2d_{0}-\sigma -\gamma_{1}\}=\frac{\dot{S}}{S}-\frac{\dot{L}}{L}-2d_{0}-\gamma_{1}-min\left\{\gamma_{3},\sigma \right\}$, $∥B_{12})∥=\frac{1}{N}\beta S$ and ‖*B*_21_)‖ = *max*{*σ*, 0} = *σ*. Therefore *g*_1_ and *g*_2_ becomes, such that, $g_{1}\leq \frac{\dot{S}}{S}-2d_{0}-\gamma_{3}-\sigma$ and $g_{2}\leq \frac{\dot{S}}{S}-2d_{0}-\gamma_{1}-min\left\{\gamma_{3},\sigma \right\}+\sigma$, which implies that $\ell \left(B\right)\leq \{\frac{\dot{S}}{S}-2d_{0}-min\left\{\gamma_{3},\sigma \right\}+\sigma \}.$ Hence $\ell \left(B\right)\leq \frac{\dot{S}}{S}-2\mu_{0}$. Now integrating the Lozinski measure *ℓ*(*B*) with respect to *t* in the interval [0, *t*] and taking lim_*t*→∞_, we obtain

$$\begin{array}{c} \lim_{t\to \infty }\text{s}up\;sup\frac{1}{t}\int_{0}^{t}\ell \left(B\right)dt<-2\mu_{0}. \end{array}$$ (33)

So finally, we can write

$$\bar{q}=\lim_{t\to \infty }sup\;sup\frac{1}{t}\int_{0}^{t}\ell \left(B\right)dt<0.$$

Thus the system containing the first three equations of the model (1) is globally asymptotically stable around its interior equilibrium (S_*_, L_*_, A_*_). Further more the solution of the limiting system of the remanning subsystem gives that *C*(*t*) → C_*_ and *V*(*t*) → V_*_. Hence F_*_ = (S_*_, L_*_, A_*_, C_*_, R_*_, V_*_) is globally asymptotically stable.

## Numerical simulation

In this section, the numerical simulations of the proposed model (1) are presented for the verification of analytical results. The numerical results are obtained by using the Runge-Kutta method of order four. The parameters value used in the simulation are given in Table 2, which are biologically feasible.

**Table 2:** Parameter values used in numerical simulation.

Parameter	Parameter description	value
*b*	Birth rate	0.0121
*ξ*	Birth rate without successful vaccination	0.0500
*η*	Proportion of parentally infected individuals	0.0110
*ϕ*	Rate of waning vaccine induced immunity	0.1000
*σ*	Moving rate from latent class to acute class	0.0012
*β*	Hepatitis B transmission rate	0.0950
*γ*_1_	Moving rate from acute to chronic carrier	0.3300
*γ*_2_	Moving rate from chronic carrier to immune	0.0090
*γ*_3_	Vaccination rate	0.0200
*d*_0_	Natural mortality rate	0.0121
*d*_1_	Hepatitis B induced death rate	0.0026
*p*	Probability of fails individual, who recovers in acute stage	0.7000
*S*(*t*)	Susceptible population	800–1000
*L*(*t*)	Latent population	200–400.0
*A*(*t*)	Acutely infected population	100–300.0
*C*(*t*)	Chronically infected population	50.0–200.0
*R*(*t*)	Recovered population	20.0–100.0
*V*(*t*)	Vaccinated population	20.0–100.0

**Fig 13:**
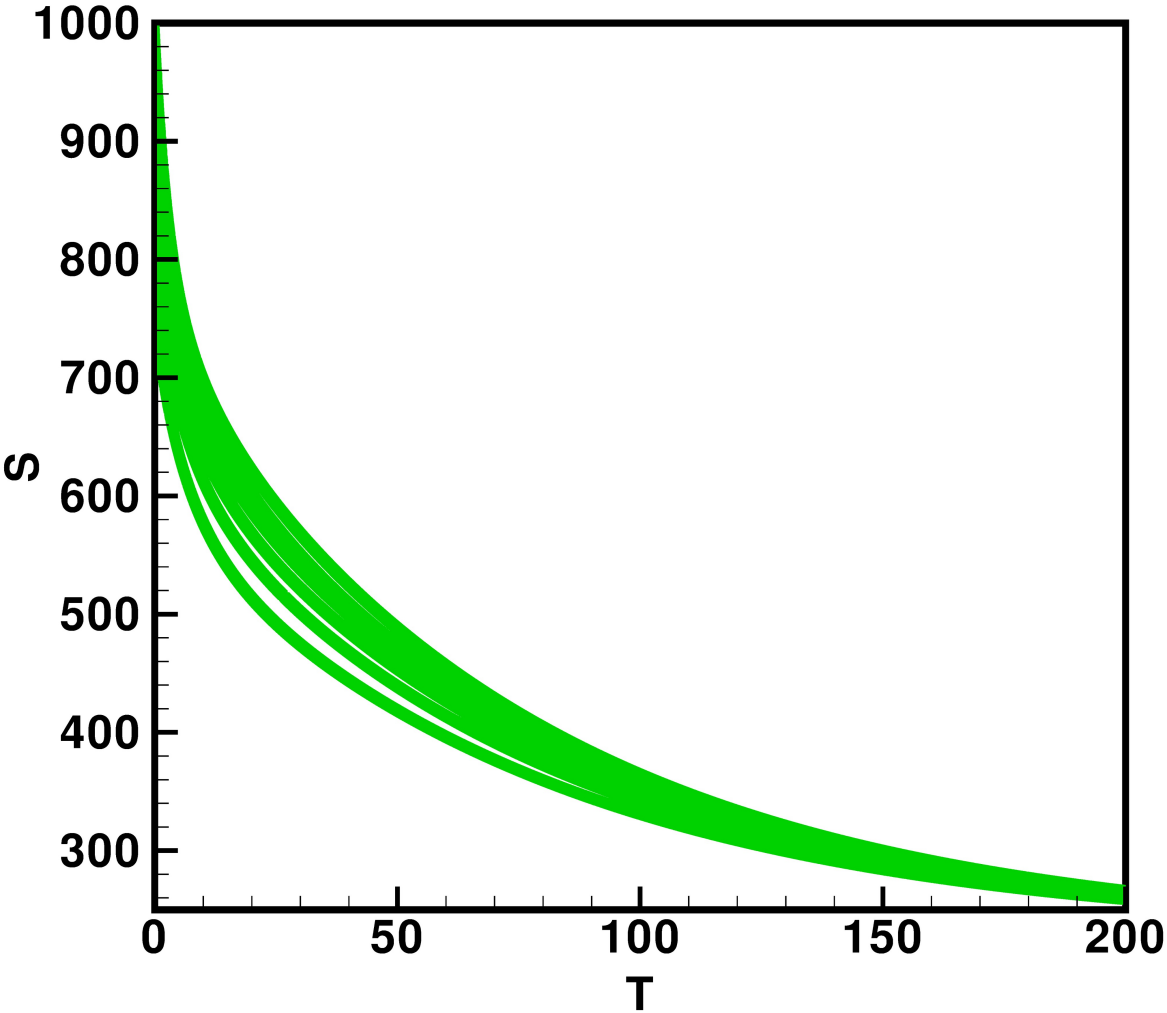
The plot represents the time dynamics of the susceptible population.

**Fig 14:**
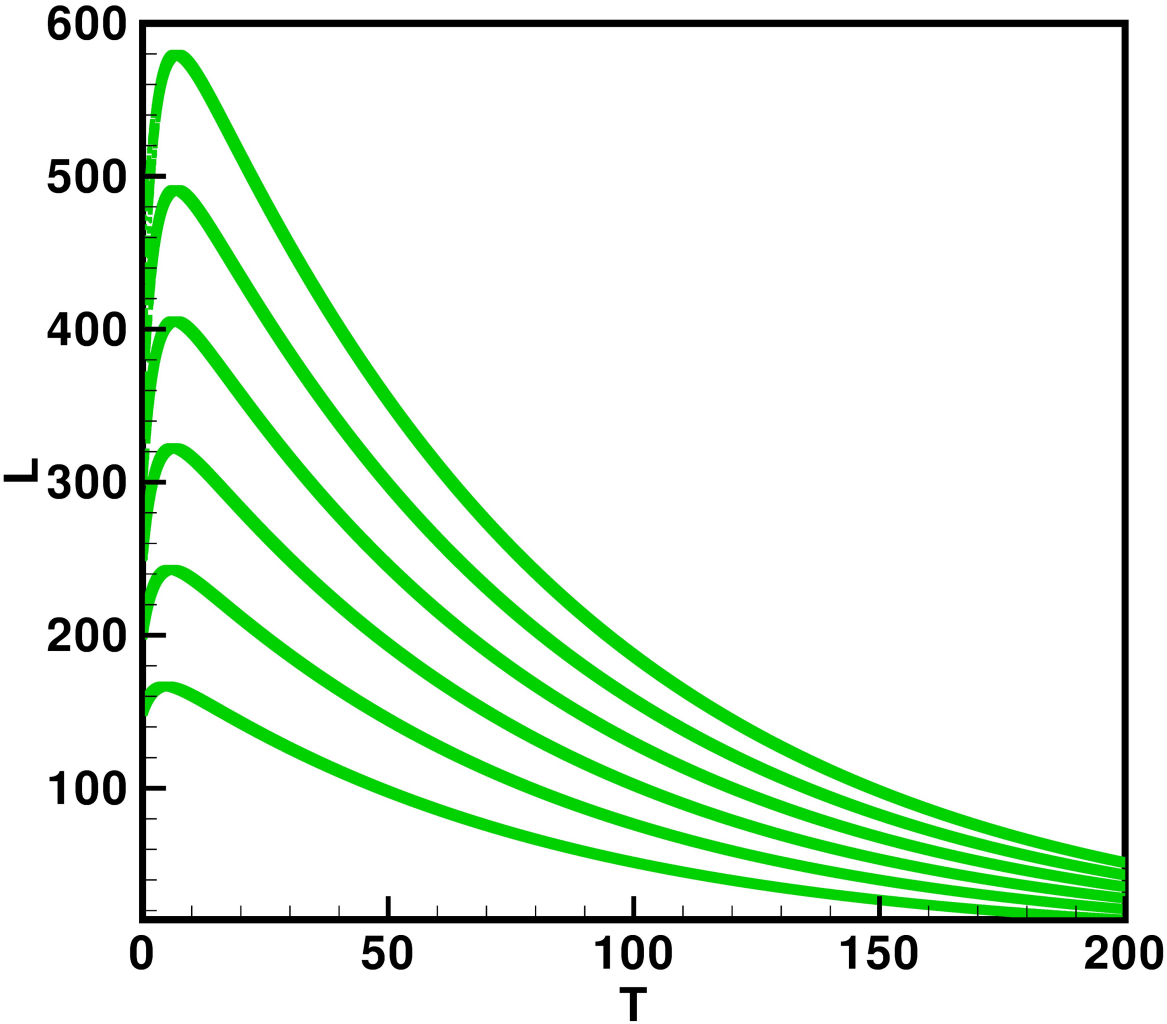
The plot represents the time dynamics of the latent population.

**Fig 15:**
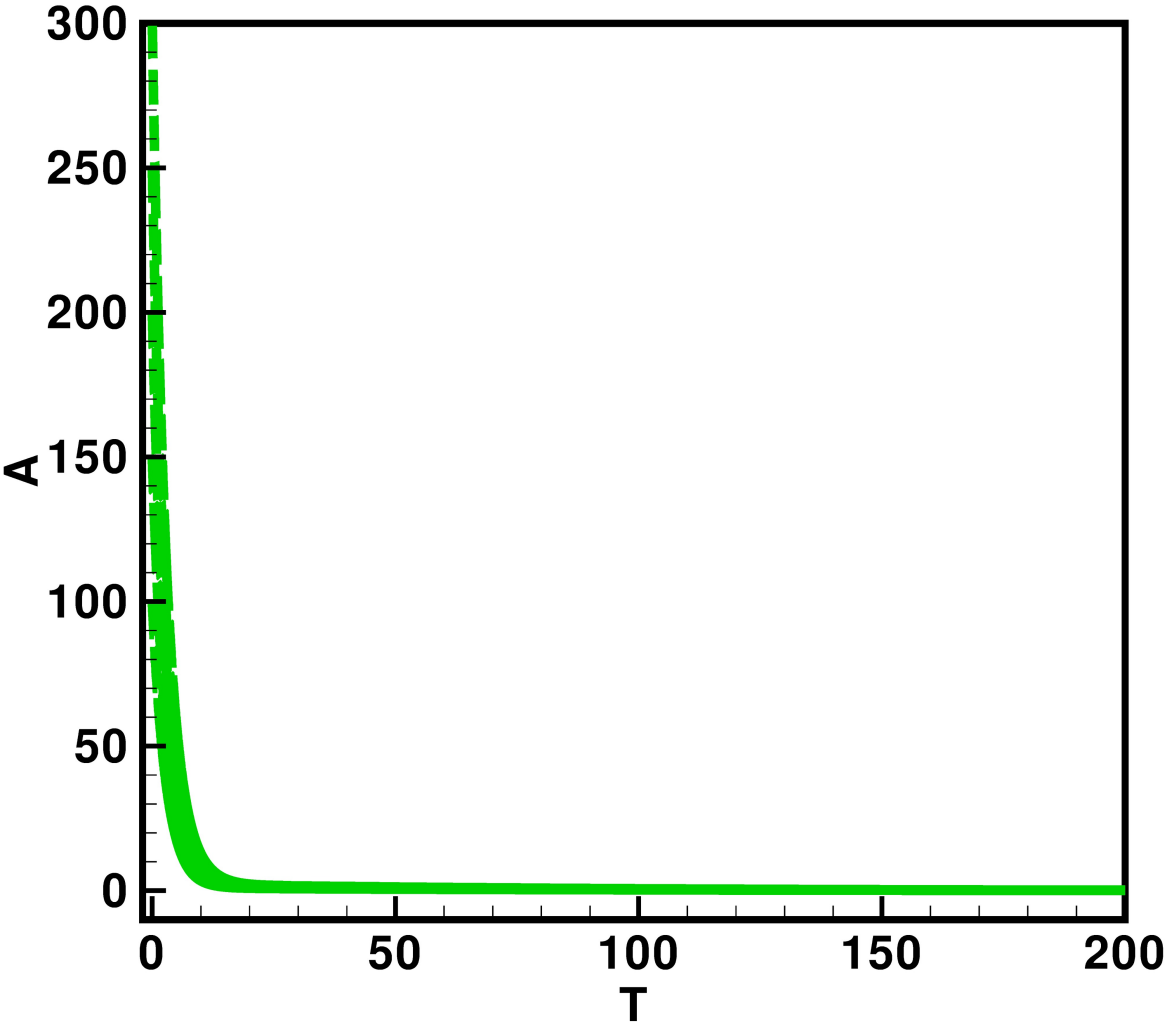
The plot represents the time dynamics of the acutely infected population.

**Fig 16:**
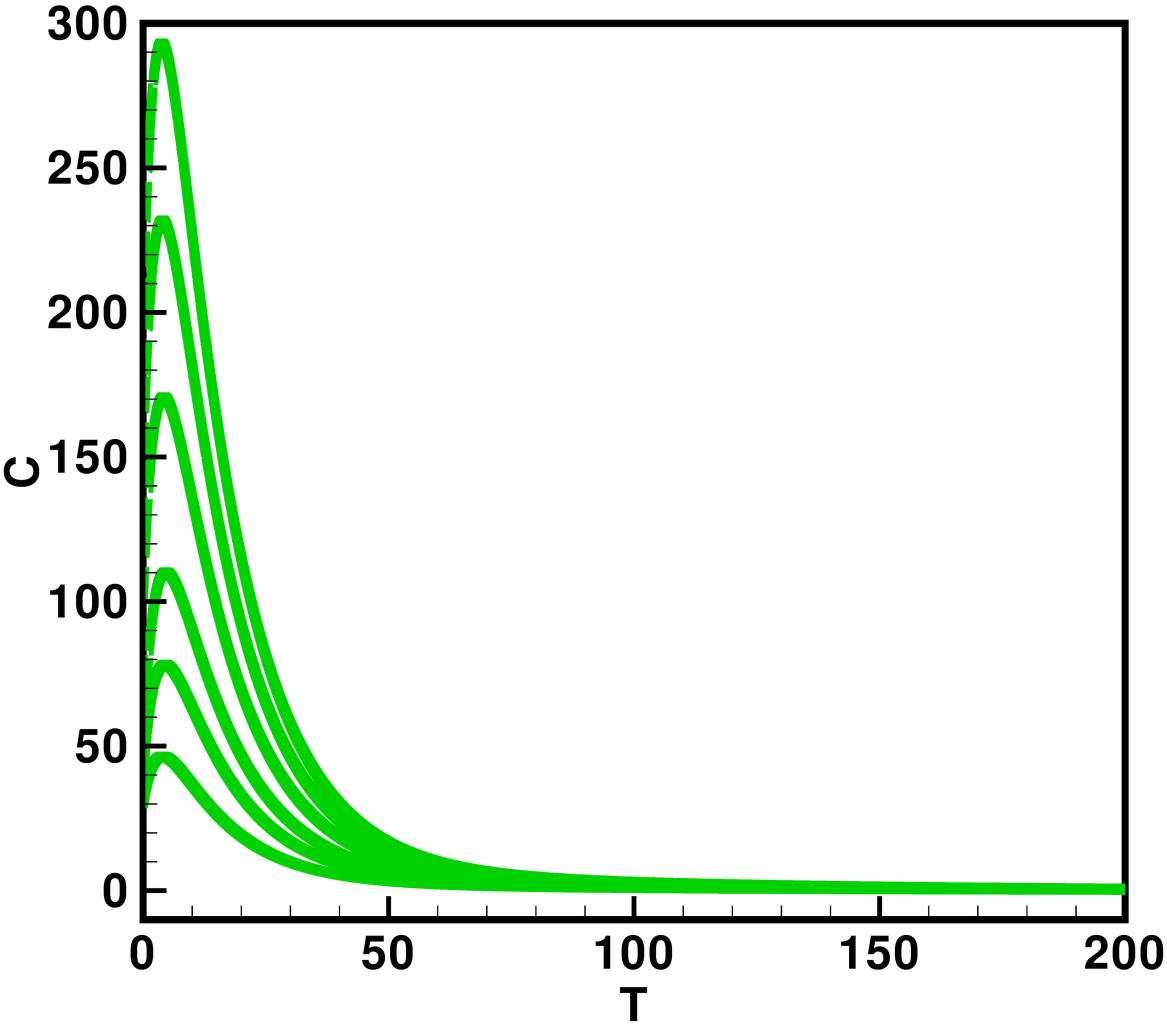
The plot represents the time dynamics of the chronic carrier population.

**Fig 17:**
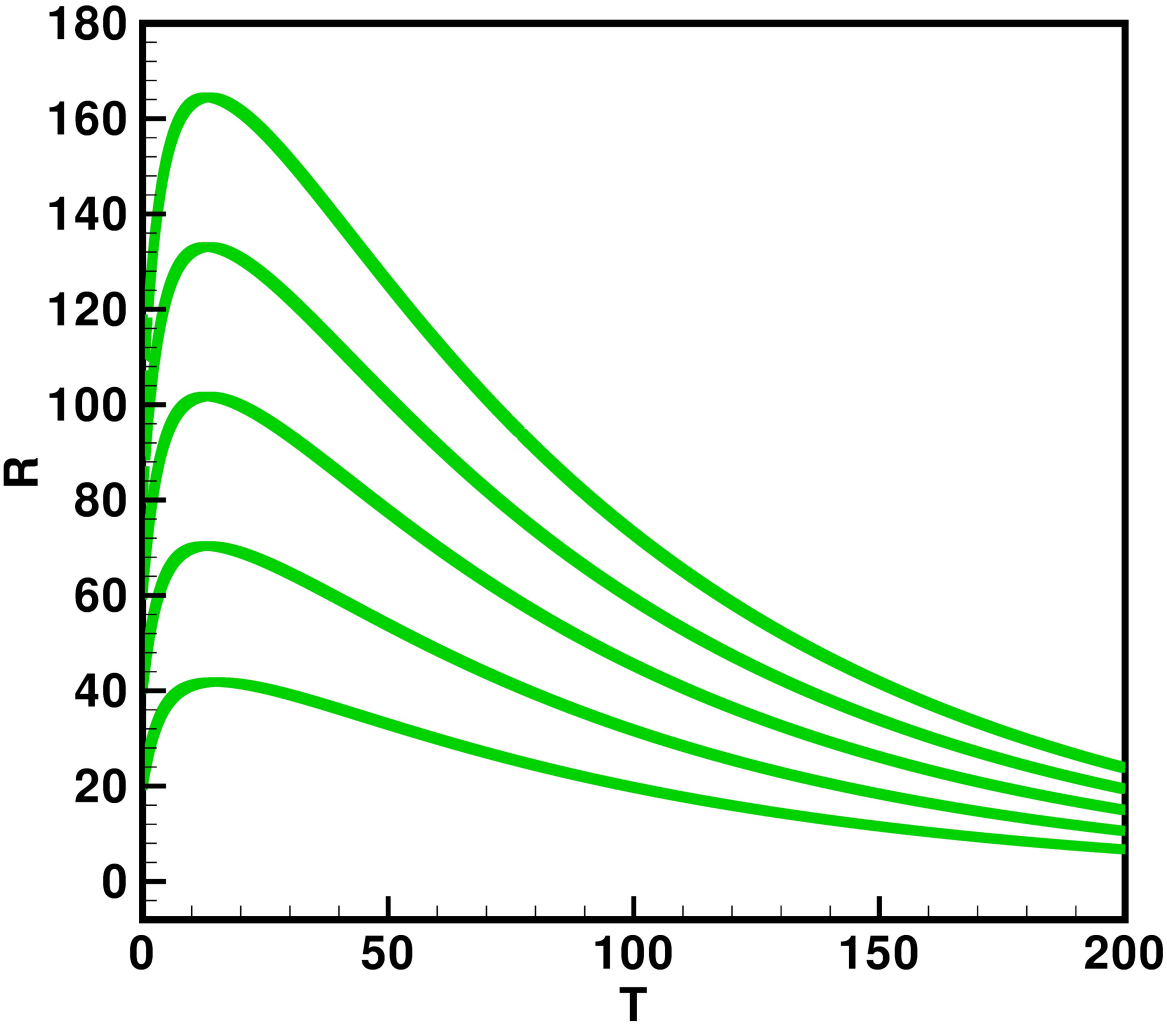
The plot represents the time dynamics of the recovered population.

**Fig 18:**
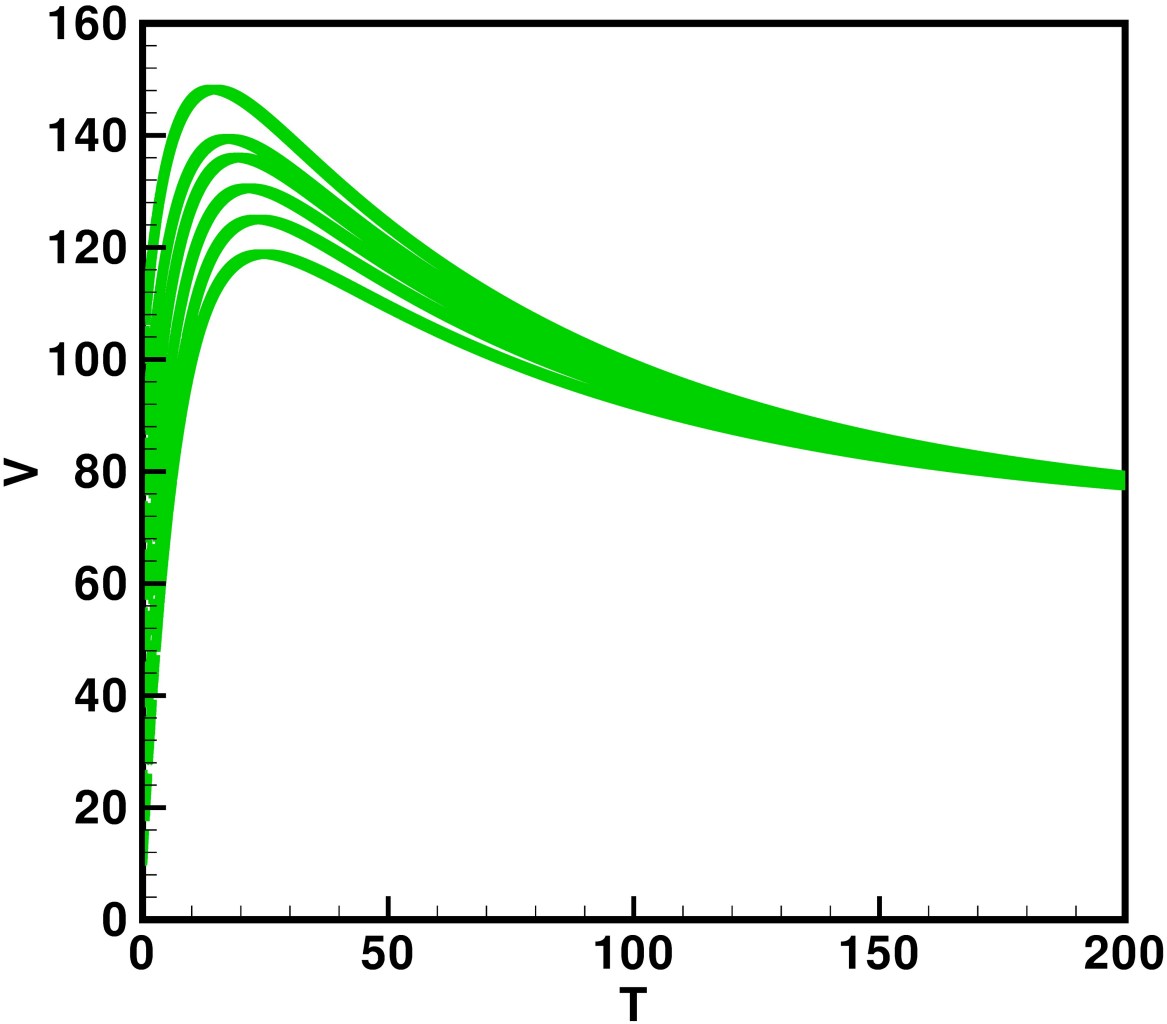
The plot represents the time dynamics of the vaccinated population.

Moreover the time interval is taken 0–200 units, while the different initial population size for the compartmental population susceptible *S*(*t*), latent *L*(*t*), acutely infected *A*(*t*), chronic carries *C*(*t*), recovered *R*(*t*) and vaccinated individuals *V*(*t*) are presented in Table 2. By using the parameters value, non-negative initial population sizes and the time interval 0–200, we obtain the simulation Figs (13) to (18), which represents that there are always susceptible *S*(*t*) and vaccinated *V*(*t*) population, while the remaining individuals i.e., acutely infected *A*(*t*), chronic carrier *C*(*t*) and recovered *R*(*t*) vanishes. The trajectories of susceptible population *S*(*t*), latent population *L*(*t*), acutely infected population *A*(*t*), chronic carrier population *C*(*t*), recovered population *R*(*t*) and vaccinated population *V*(*t*) converges to the equilibrium points. Which ensure the stability of the proposed model. It is also be noted that our proposed model shows that the susceptible and acutely infected individuals are decreasing sharply, while the latent, chronic carrier, recovered and vaccinated individuals are increasing at the beginning and then decreasing after some time as shown in Figs (13) to (18).

## Conclusion and discussion

In this article, we have established a model for the transmission dynamic of hepatitis B with nonlinear incidence by taking into account the classification of different phases of hepatitis B (acutely and chronically) septic individuals. We presented different mathematical analysis including positivity, boundedness and biological feasibility of the proposed model. We obtained the basic reproduction number by using the next generation matrix approach and then discussed its sensitivity analysis by normalized sensitivity index. Moreover, we discussed the stability analysis and showed that the proposed model is both locally as well as globally asymptotically stable for the disease free as well as for endemic equilibriums. For the local stability, linearization and Routh-Herwitz criteria have been used, while the global stability is retrieved by using Lyapunov function theory and geometrical approach. Finally, the numerical simulation and sensitivity analysis are presented to show the feasibility of the proposed work.

In future, we will consider the proposed model with spatial effect. We will also design the optimal control strategy on the basis of normalized sensitivity index of basic reproduction number to minimize the number of infected hepatitis B individuals and to maximize the number of noninfected individuals. Work on such issues are in progress and will be reported in a near future publication.
